# Reliability and validity of a new accelerometer-based device for detecting physical activities and energy expenditure

**DOI:** 10.7717/peerj.5775

**Published:** 2018-10-11

**Authors:** Yanxiang Yang, Moritz Schumann, Shenglong Le, Shulin Cheng

**Affiliations:** 1 Department of Physical Education, Exercise, Health and Technology Center, Shanghai Jiao Tong University, Shanghai, China; 2 Institute of Cardiovascular Research and Sport Medicine, Department of Molecular and Cellular Sport Medicine, German Sport University, Cologne, Germany; 3 The Exercise Translational Medicine Center, Shanghai Jiao Tong University, Shanghai, China; 4 Unit of Health Sciences, Faculty of Sport and Health Sciences, University of Jyväskylä, Jyväskylä, Finland; 5 The Key Laboratory of Systems Biomedicine, Ministry of Education, Shanghai Center for Systems Biomedicine, Shanghai Jiao Tong University, Shanghai, China

**Keywords:** Sedentary behavior, Motion, Posture allocation, Activity tracker

## Abstract

**Background:**

Objective assessments of sedentary behavior and physical activity (PA) by using accelerometer-based wearable devices are ever expanding, given their importance in the global context of health maintenance. This study aimed to determine the reliability and validity of a new accelerometer-based analyzer (Fibion) for detecting different PAs and estimating energy expenditure (EE) during a simulated free-living day.

**Methods:**

The study consisted of two parts: a reliability (*n* = 18) and a validity (*n* = 19) test. Reliability was assessed by a 45 min protocol of repeated sitting, standing, and walking (i.e., 3 × 15 min, repeated twice), using both Fibion and ActiGraph. Validity was assessed by a 12 h continuous sequence tasks of different types (sitting, standing, walking, and cycling) and intensities (light [LPA], moderate [MPA], and vigorous [VPA]) of PA. Two Fibion devices were worn on the thigh (FT) and in the pocket (FP), respectively and were compared with criteria measures, such as direct observation (criterion 1) and oxygen consumption by a portable gas analyzer, K4b^2^ (criterion 2).

**Results:**

FT (intra-class correlation coefficients (ICCs): 0.687–0.806) provided similar reliability as the Actigraph (ICCs: 0.661–0.806) for EE estimation. However, the measurement error (ME) of FT compared to the actual time records indicated an underestimation of duration by 5.1 ± 1.2%, 3.8 ± 0.3% and 14.9 ± 2.6% during sitting, walking, and standing, respectively. During the validity test, FT but not FP showed a moderate agreement but lager variance with the criteria (1 and 2) in assessing duration of sitting, long sitting, LPA, MPA, and VPA (*p* > 0.05, ICCs: 0.071–0.537), as well as for EE estimation of standing, LPA, MPA, and VPA (*p* > 0.05, ICCs: 0.673–0.894).

**Conclusions:**

FT provided similar reliability to that of the Actigraph. However, low correlations between subsequent measurements of both devices indicated large random MEs, which were somewhat diminished during the simulated 12 h real-life test. Furthermore, FT may accurately determine the types, intensities of PA and EE during prolonged periods with substantial changes in postures, indicating that the location of the accelerometer is essential. Further study with a large cohort is needed to confirm the usability of Fibion, especially for detecting the low-intensity PAs.

## Introduction

Assessment of sedentary behavior and physical activity (PA) is ever expanding, given its importance in the global context of health maintenance (Ainsworth et al., 2011a; Hills, Mokhtar & Byrne, 2014). Especially, accelerometer-based wearable devices have become increasingly popular over the past decade. However, the different algorithms and output parameters make it difficult to determine their accuracy. Furthermore, the function and accuracy of the devices may significantly differ between the wearing locations (Loprinzi & Smith, 2017). For example, wrist-worn devices such as Fitbit and Jawbone have been shown to provide higher accuracy of steps but lower validity for EE, compared to indirect or direct calorimetry, accelerometry, and self-reported EE (Evenson, Goto & Furberg, 2015). In addition, waist- or thigh-based accelerometers, such as the ActivPAL monitor were designed to differentiate sitting/supine postures from standing, while it may not differentiate PA types (Steeves et al., 2015). Similarly, the IDEEA monitor can distinguish between 32 types of postures and gaits, and may also provide step counts and EE, but the complexity of the setup makes the device unfeasible for a wider use (Maffiuletti et al., 2008; Jiang & Larson, 2013).

Fibion (Fibion Inc, Jyväskylä, Finland) is a new three-axial lightweight (20 g, L × W × T = 30 × 32 × 10 mm) accelerometer-based device which was designed to follow orientation and movement of the thigh. Thus, it can be worn either on the thigh (FT) or Fibion worn in the Pocket of the trousers (FP). According to information provided by the manufacturer, it is able to detect no-wear time, to differentiate PA types (sitting, long sitting, standing, walking, and cycling) and intensities (light PA (LPA), moderate PA (MPA), and vigorous PA (VPA), as well as the associated EE. However, to the best of our knowledge, no objective data on its reliability and validity have been studied. Therefore, the aim of this study was to (1) assess the reliability of Fibion (FT) in a laboratory-based test-retest protocol, with comparison to ActiGraph (GT9X Link, Pensacola, FL, USA); (2) determine the validity of Fibion worn at two different locations (FT and FP) in differentiating PAs and estimating EE throughout a simulated 12 h free-living day.

## Materials and Methods

### Study design

The study included two protocols: investigating the reliability (*n* = 18) and validity (*n* = 19) of the Fibion accelerometer. All 37 participants were young and healthy volunteers, who had normal weight (i.e., BMI < 25 kg/m^2^) and were recreationally physically active. Exclusion criteria included acute and chronic diseases, which would prevent participants from prolonged sitting and/or standing or would interfere with the basic metabolic rate. All participants were informed of the study procedures and provided written informed consent prior to the testing. The study was carried out in accordance with the Declaration of Helsinki and was approved by the Ethical Committee of Shanghai Jiao Tong University (Approval number: ML16027).

### Reliability protocol

A total of 18 young adults (eight females, 10 males; age 24.0 ± 2.8 years; BMI 22.8 ± 2.3 kg/m^2^) participated in the repeatability study. The protocol is a designed 45 min repeated test of walking, standing, and sitting, respectively (3 × 15 min, repeated twice, a total of 90 min). The participants were required to simultaneously wear Fibion and ActiGraph proximally on the left thigh, between the knee and the hip (Fig. S2A). However, the exact wearing location (i.e., medial or lateral) of each device was randomized but equally distributed among all participants. The ActiGraph was chosen because it is widely used and its validity and reliability have previously been studied (Welk, Schaben & Morrow, 2004; Esliger & Tremblay, 2006; Vanhelst et al., 2012; Lee, Kim & Welk, 2014; Aadland & Ylvisåker, 2015). Throughout the data recording, the participants were instructed to sit or stand still without shuffling their feet or repositioning their body, under observation by a research assistant. Walking was performed at a self-selected constant velocity. The procedure was strictly confined to the duration of 15 min, while any deviations from the protocol (i.e., uncommanded change of postures) were recorded.

### Validity protocol

The validity protocol was performed by 19 young adults (9 females, 10 males; age 28.2 ± 3.8 years; BMI 21.0 ± 2.2 kg/m^2^), by using a 12 h guided sequence of tasks in the laboratory that simulated realistic daily activities. The participants were required to wear two Fibion devices (Fibion strapped to the front Thigh (FT) and worn in the pocket (FP)), as well as a portable gas analyzer (Cosmed K4b^2^; Cosmed, Rome, Italy) (Fig. S2B).

All participants were required to abstain from caffeine, alcohol intake, and unaccustomed exercise for 24 h prior to the measurements. The protocol commenced after resting metabolic rate was assessed in a fasting state following best practice guidelines (Compher et al., 2006). One supervisor and two assistants directly followed the participants in 4 h shifts. Specifically, the validity of the Fibion accelerometers was assessed using two criteria:

### Criterion 1: 12 h guided sequence of tasks with direct observation

Direct observation of the designed 12 h guided sequence of tasks served as the criterion for detecting different types and intensities of PA (Table S1). The direct observation has been previously proven as a valid method, compared to indirect calorimetry (Lyden et al., 2014). In the present study, the 12 h guided sequence of tasks was designed to simulate an “ideal active working day,” according to current recommendations (Buckley et al., 2015). Thus, it included both computer-based office works (i.e., sitting, standing, walking, and cycling, etc.) and leisure activities in the afternoon (i.e., actually watching TV sitting on a sofa and exercising). In particular, walking was performed either within the laboratory or the hallway (short and long walks) or on the indoor track. Cycling was carried out on an ergometer at a self-paced intensity, which allowed participants to change the cadence as they do in the real-life conditions (i.e., commuting to/from work).

For the data collection, each of the tasks was coded as 0–12 based on the measurement logs of the direct observations, as was done previously (Lyden et al., 2017). The codes were subsequently separated into five different types (sitting, long sitting, standing, walking, and cycling) and three intensities (LPA, MPA, and VPA) of PA (Table S2). On the basis of the metabolic equivalent (MET; Ainsworth et al., 2011b), the PA intensities were defined as follows: long sitting was defined as uninterrupted sitting periods over 30 min. LPA was defined as any activities with an EE below three METs, excluding sitting. MPA was defined as activities with an EE between three and six METs. VPA was defined as activities over six METs. The entire 12 h measurement was recorded by video, to verify the correct timing of each criterion.

### Criterion 2: indirect calorimetry

Indirect calorimetry served as the criterion for EE estimation. Pulmonary gas exchange of the participants was continuously measured throughout the 12 h guided sequence of tasks by a portable breath-by-breath gas analyzer (Cosmed K4b^2^, Rome, Italy). The K4b^2^ has previously been shown to be a valid and reliable device for estimating oxygen consumption (McLaughlin et al., 2001; Schrack, Simonsick & Ferrucci, 2010). Each participant was fitted with a rubber facemask (Hans-Rudolph, Kansas City, MO, USA), while the gas sensor was attached to a harvest and carried on the chest throughout the protocol (Fig. S2B). Prior to data recording, the device was calibrated according to the manufacturer’s guidelines (approximately 10 min before the start of the measurement). The facemask was removed from the face only during designated short breaks, that is, for food intake.

### Data processing

Fibion data collected from the reliability and validity measurements were uploaded to the manufacturer’s web-browser-based online service (www.fibion.com/upload), in order to obtain detailed reports on PA types and intensities as well as the corresponding EE. The raw comma-separated values (CSV) files with minute-by-minute data from Fibion were subsequently exported for further analysis. Similarly, the obtained ActiGraph data were uploaded to the device-specific software (ActiLife 6) and subsequently exported to Microsoft Excel for further analyses. The ActiGraph data for each activity type (provided in seconds) were then synchronized with manual recordings. To obtain comparable data of EE, the breath-by-breath values collected with the K4b^2^ were averaged for the duration of each task and were expressed as average “kcals/min” of each PA type and a 12 h total EE (Tables S1 and S2). No data were removed from the reliability study. However, for the validity test, a total of 8.2 ± 2.3% of the data, both from Fibion and criteria, were excluded due to visible artifacts (i.e., originating from movement artifacts, errors in the assessment of EE by gas analysis, or meal breaks). Furthermore, validity data from two participants (ID 16, 17) of FT but not FP were removed entirely due to wrong positioning of the thigh strap, according to the retrospective check of the video recordings.

### Statistical analyses

#### Reliability

The measurement precision of Fibion and ActiGraph was expressed as the coefficient of variation (“CV% = (RMS − SD)/mean × 100,” where the “RMS-SD” refers to the root mean square (RMS) of the standard deviation (SD) calculated from both measurements of each individual (i.e., for sitting, standing, and walking, respectively) and “mean” refers to the group mean (Brunzendorf & Behrens, 2007; Finni et al., 2007)). The CV% was classified as low (<10%), medium (10–20%), and high (20–30%) (Gomes, 2009). In addition, percent measurement error (ME) was calculated by the equation: “ME = (actual duration/estimated duration) × 100–100,” where the actual duration for each type was 15 min. Intra-class correlation coefficient (ICC) two-way ANOVA models were used to determine the random/individual errors between tests. ICC may be interpreted as low (<0.4), moderate (0.4–0.75), and high (>0.75), respectively (Cicchetti, 1994). The paired samples Student’s *t*-test was used to compare the difference between Fibion and ActiGraph. If data were not normally distributed even after log transformation, the Wilcoxon signed-rank test was used.

#### Validity

Paired difference tests (Student’s *t*-tests or Wilcoxon signed-rank test) and the ICC were used to determine the accuracy of Fibion compared to criterion 1 and 2. Bland–Altman plots were generated to examine the mean bias and limits of agreement (LOA, mean difference ±1.96 SD) of Fibion in comparison to the criteria for each PA type, intensity, and the 12 h total EE (Bland & Altman, 1986). All statistical analyses were performed with the R program (RStudio Team, 2018), and the level of significance was set at *p* < 0.05 (two-sided).

## Results

### Reliability

Table 1 presents the mean ± SD (ME, only for PA duration) of the repeated performances of Fibion (FT) and ActiGraph. The agreement of FT was moderate to good (CV%: 6.57–9.13; ICCs: 0.687–0.806) in all EE measures of sitting, standing, and walking, similar to the ActiGraph. FT accurately detected the duration of sitting (ME: 5.1 ± 1.2%), and walking (ME: 3.8 ± 0.3%), but not for standing (ME: 14.9 ± 2.6%). However, low correlations (ICCs: 0.189–0.459) were observed between subsequent measurements with both devices, especially in low-intensity PAs (sitting).

**Table 1:** Mean and standard deviation (%ME, only for PA duration) of repeatability measurements for Fibion worn on the thigh (FT) and ActiGraph (95% Confidence Interval).

		Sitting		Standing		Walking	
		FT	ActiGraph	FT	ActiGraph	FT	ActiGraph
AT	1st test ME	14.40 ± 0.30 (4.22)	14.09 ± 1.26 (7.40)	13.36 ± 1.03 (13.00)	13.83 ± 1.12 (9.20)	14.45 ± 0.45 (4.00)	14.86 ± 0.78 (3.8)
	2nd test ME	14.18 ± 0.33 (5.85)	13.98 ± 0.95 (8.20)	12.98 ± 0.90 (16.07)	13.89 ± 1.41 (11.00)	14.48 ± 0.39 (3.60)	14.60 ± 0.88 (5.20)
	CV%	2.19	7.28	5.51	5.68	2.54	6.08
	*p*	**0.031**	0.587	0.112	0.486	0.760	0.390
	ICC	0.189	0.119	0.459	0.609	0.227	−0.165
EE	1st test	1.28 ± 0.201	1.30 ± 0.211	1.73 ± 0.23	1.74 ± 0.25	3.40 ± 0.61	3.52 ± 0.54
	2nd test	1.33 ± 0.18	1.38 ± 0.21	1.70 ± 0.25	1.76 ± 0.24	3.44 ± 0.73	3.47 ± 0.63
	CV%	6.57	7.14	7.79	7.44	9.13	9.69
	*p*	0.081	**0.014**	0.058	0.523	0.551	0.766
	ICC	0.806	0.806	0.687	0.789	0.782	0.661

**Note:**

AT, refers to the duration of activity types; EE, refers to the energy expenditure; CV%, coefficient of variation; ME, percent measurement error; ICC, intra-class correlation coefficient; *p*-values refer to the Student’s *t*-test or the Wilcoxon signed-rank test.

The bold *p*-values indicating significant differences between the compared two groups.

### Validity

Table 2 presents the mean and SD of different PA durations (min) and EE (kcal/min) of Fibion compared with criterion 1 and 2.

**Table 2:** Mean and standard deviation of different PAs detection (time duration, min), specified-types of EE (kcal/min), and a 12 h total EE (kcals) measured with Fibion devices (FT and FP), compared to criterion 1 (direct observation) and criterion 2 (K4b^2^). (95% Confidence Interval).

		Criterion 1 *n* = 17	FT *n* = 17	ICC	*p*-Value	Criterion 1 *n* = 19	FP *n* = 19	ICC	*p*-Value	*p-Value *n* = 17
PA types/intensities detection (min)	Sitting	491.7 ± 10.2	487.5 ± 22.4	0.407	0.329	492.0 ± 9.7	411.4 ± 115.5	−0.046	**0.009**	0.051
	LSit	292.0 ± 20.1	283.0 ± 56.9	0.220	0.501	291.8 ± 19.0	199.6 ± 123.4	0.060	**0.012**	**0.041**
	Standing	80.1 ± 7.6	97.3 ± 17.6	0.204	**<0.000**	80.3 ± 7.2	178.6 ± 121.9	−0.036	**<0.000**	**0.040**
	Walking	88.5 ± 10.7	71.2 ± 13.2	0.092	**<0.000**	90.1 ± 11.2	74.7 ± 16.1	0.154	**<0.000**	0.306
	Cycling	29.8 ± 0.5	23.0 ± 6.1	0.016	**<0.000**	29.8 ± 0.5	17.5 ± 12.1	−0.011	**<0.000**	0.207
	LPA	158.0 ± 15.9	154.0 ± 13.8	0.537	0.240	160.3 ± 17.6	160.7 ± 16.4	0.650	0.900	**0.009**
	MPA	85.4 ± 28.7	76.8 ± 11.1	0.276	0.266	82.6 ± 30.0	59.1 ± 16.0	0.220	**0.002**	**<0.000**
	VPA	18.9 ± 13.4	15.1 ± 7.6	0.071	0.320	18.5 ± 12.8	11.8 ± 6.4	0.376	**0.014**	0.159
	TD	700.0 ± 15.3	694.0 ± 20.7	0.638	0.121	702.1 ± 15.7	694.0 ± 18.6	0.534	**0.055**	0.854

		Criterion 2 *n* = 17	FT *n* = 17	ICC	*p-*value	Criterion 2 *n* = 19	FP *n* = 19	ICC	*p*-value	*p-value *n* = 17
EE estimation (kcal/min)	Sitting	1.5 ± 0.4	1.4 ± 0.3	0.647	**0.039**	1.6 ± 0.4	1.4 ± 0.3	0.674	**0.045**	0.584
	Standing	1.9 ± 0.4	1.8 ± 0.3	0.708	0.258	1.8 ± 0.4	1.8 ± 0.3	0.765	0.149	0.363
	Walking	2.9 ± 0.6	3.4 ± 0.8	0.374	**0.014**	2.8 ± 0.6	3.2 ± 0.8	0.538	**0.028**	**0.011**
	Cycling	4.7 ± 1.8	7.4 ± 1.7	0.363	**<0.000**	4.5 ± 1.8	5.5 ± 1.7	0.540	**0.009**	**<0.000**
	LPA	1.5 ± 0.2	1.5 ± 0.3	0.673	0.580	1.5 ± 0.18	1.5 ± 0.24	0.732	0.623	**0.030**
	MPA	5.6 ± 1.6	5.4 ± 1.8	0.826	0.329	5.5 ± 1.6	4.6 ± 1.3	0.624	**0.001**	**<0.000**
	VPA	7.6 ± 3.7	6.8 ± 3.8	0.894	0.083	7.5 ± 3.5	6.2 ± 3.2	0.803	**0.007**	**0.020**
	12 h total EE	1,427 ± 323.7	1,472 ± 230.7	0.743	0.377	1,396 ± 322.2	1,446 ± 216.0	0.723	0.296	0.300

**Notes:**

PA, physical activity; EE, energy expenditure; FT, Fibion worn on the thigh; FP, Fibion worn in the pocket; LSit, Long sitting (sitting periods over 30 min); LPA, light intensity PA (MET < 3, excluding sitting); MPA, moderate PA (3 < MET < 6); VPA, vigorous intensity PA (MET > 6); TD, total duration; 12 h EE, total EE during 12 h measurement protocol. ICC, Intra-class correlation coefficient; *p*-values refer to the Student’s *t*-test or the Wilcoxon signed-rank test.

* *p*-Value = FT vs. FP.

The bold *p*-values indicating significant differences between the compared two groups.

#### Criterion 1

**Figure 1:**
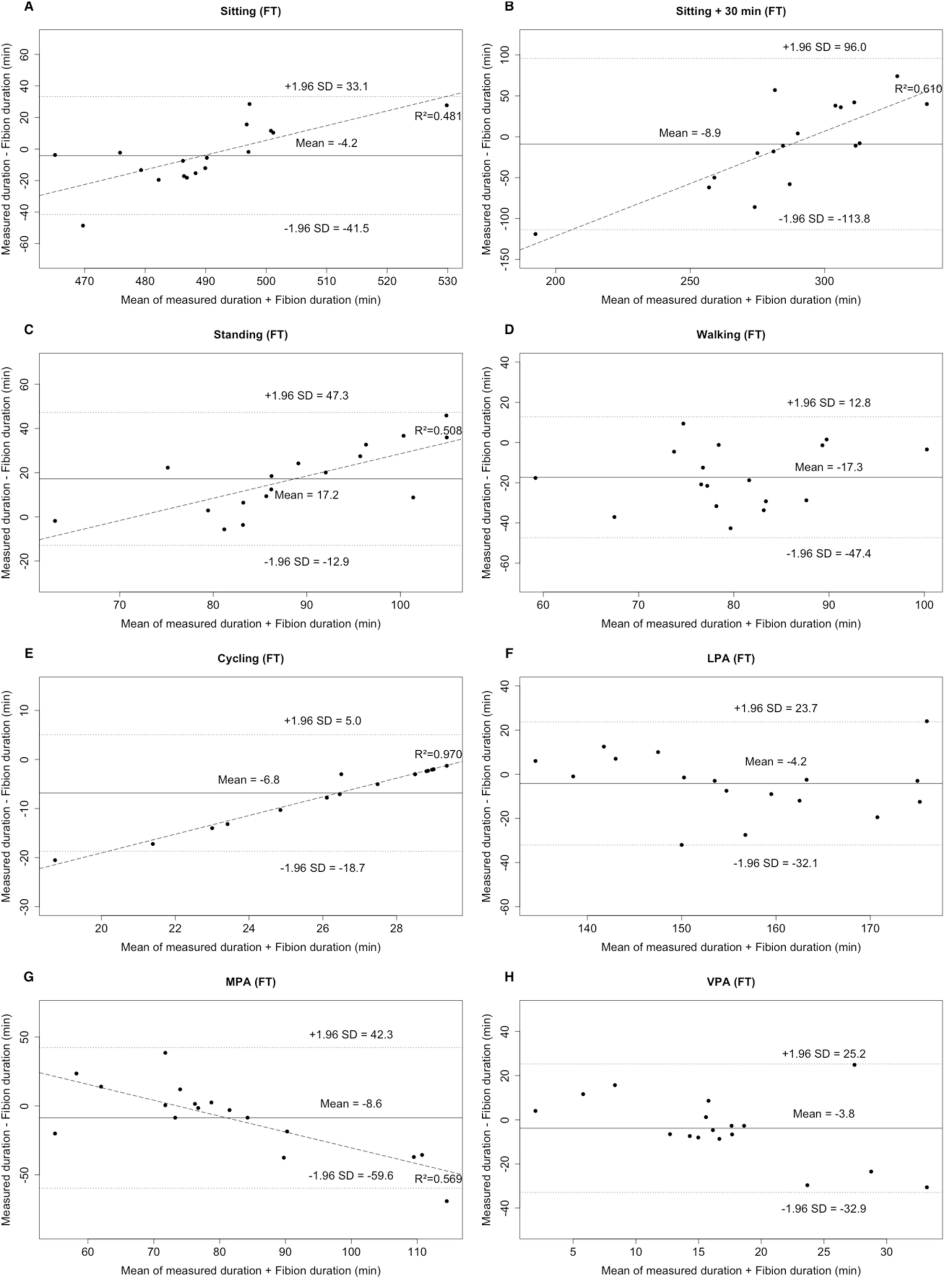
Bland–Altman plots for the duration of different PA types (A–E: sitting, long sitting, standing, walking, cycling) and intensities (F–H: LPA, MPA, VPA) for Fibion worn on the thigh (FT). The middle line shows the mean difference between FT and criterion 1 and the dashed lines indicate the limits of agreement (±1.96 × SD of the different scores). Linear regression lines were fitted for proportional error.

**Figure 2:**
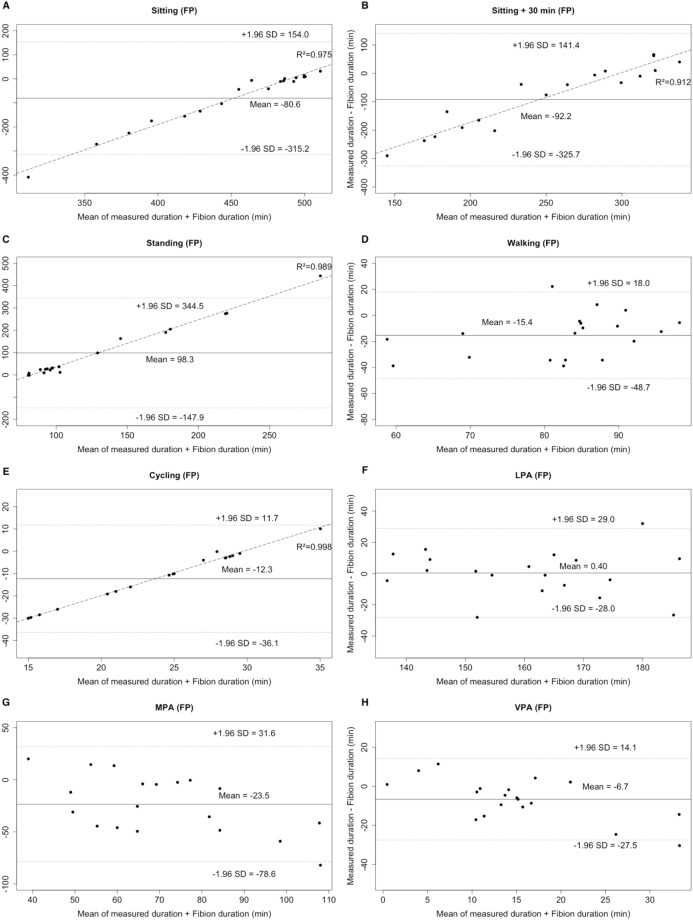
Bland–Altman plots for the duration of different PA types (A–E: sitting, long sitting, standing, walking, cycling) and intensities (F–H: LPA, MPA, VPA) for Fibion worn in the pocket (FP). The middle line shows the mean difference between FP and criterion 1 and the dashed lines indicate the limits of agreement (±1.96 × SD of the different scores). Linear regression lines were fitted for proportional error.

No significant differences were observed between FT and criterion 1 (i.e., PA duration) in sitting, long sitting, LPA, MPA, and VPA (all *p* > 0.05). By contrast, all types of FP (excepting LPA) significantly differed from criterion 1 (*p* < 0.05). When comparing FT and FP to the criterion 1, Bland–Altman plots revealed an individualized underestimation (positive mean bias) for standing and LPA, and an overestimation (negative mean bias) for all other PAs (Figs. 1 and 2). The LOA of PA durations ranged from −113.8 to 96.0 min for FT and from −325.7 to 344.5 min for FP. Furthermore, proportional errors were observed for FT (sitting, long sitting, standing, cycling, and MPA; (coefficient of determination) *R*^2^: 0.481–0.970) and FP (sitting, long sitting, and standing; *R*^2^: 0.912–0.989) and (Figs. 1 and 2). The low correlations (ICCs, FT: 0.016–0.638; FP: −0.046 to 0.650) also indicated that the MEs were random.

#### Criterion 2

**Figure 3:**
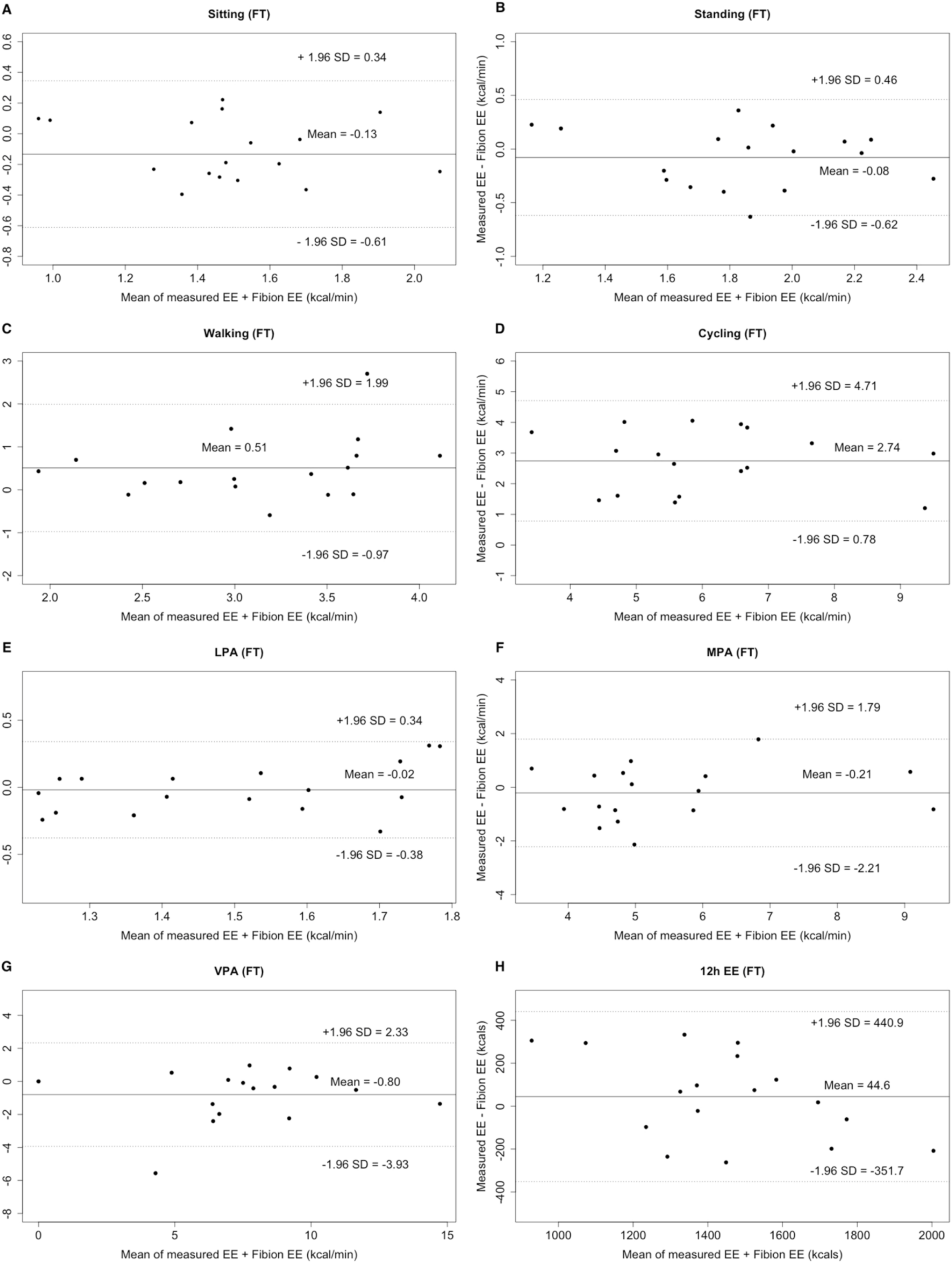
Bland–Altman plots of specified-types of EE (A–G: sitting, standing, walking, cycling, LPA, MPA, and VPA, kcal/min) and 12 h total EE (H, kcal) for Fibion worn on the thigh (FT). The middle line shows the mean difference between FT and criterion 2 and the dashed lines indicate the limits of agreement (±1.96 × SD of the different scores).

**Figure 4:**
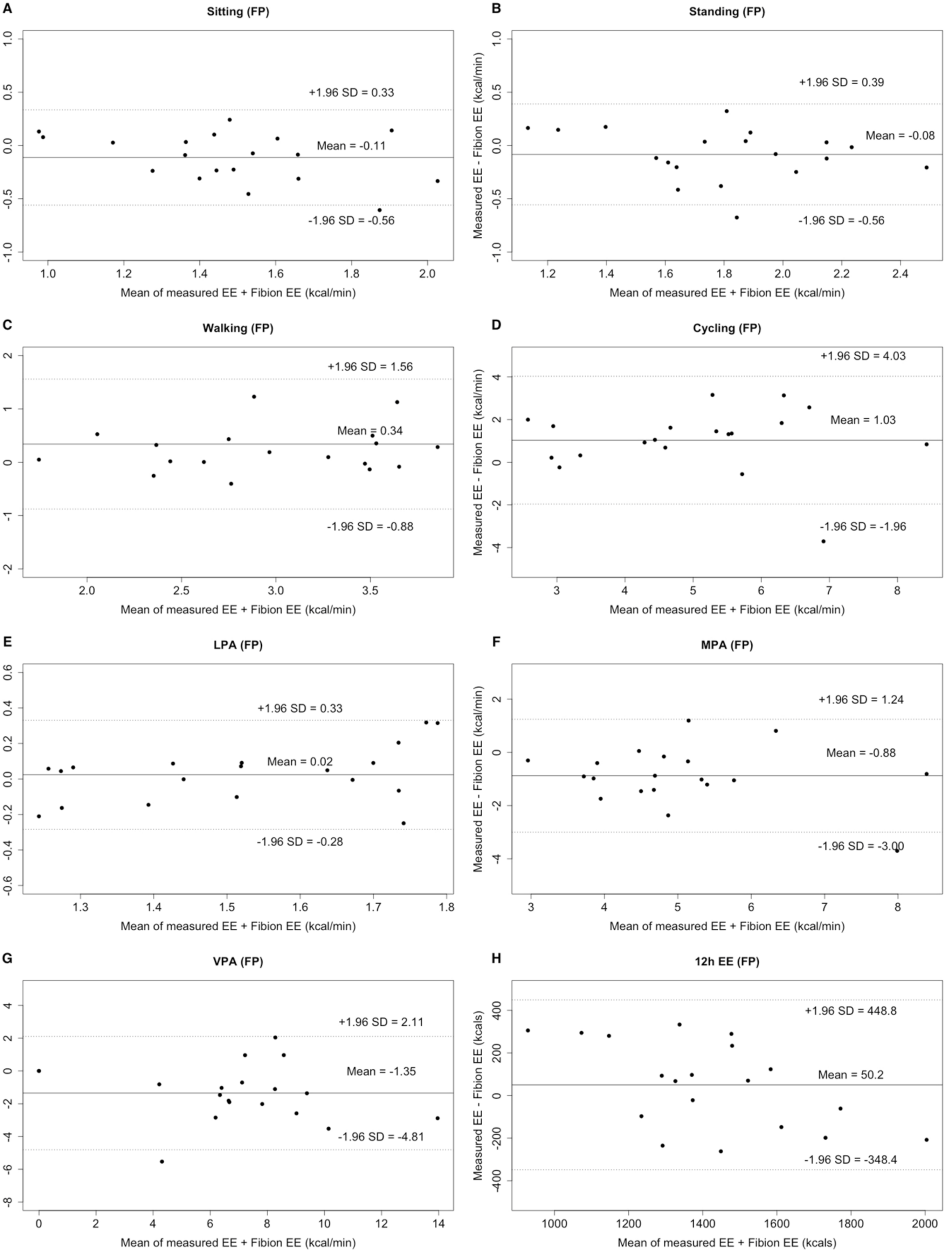
Bland–Altman plots of specified-types of EE (A–G: sitting, standing, walking, cycling, LPA, MPA, and VPA, kcal/min) and 12 h total EE (H, kcal) for Fibion worn in the pocket (FP). The middle line shows the mean difference between FP and criterion 2 and the dashed lines indicate the limits of agreement (±1.96 × SD of the different scores).

The EE of standing, LPA, MPA, VPA, as well as the overall 12 h EE did not differ between FT and criterion 2 (all *p* > 0.05; ICCs: 0.363–0.894). However, all other activities significantly differed between FP and criterion 2, except for sitting, standing, LPA, and 12 h EE (Table 2). Bland–Altman plots revealed an individualized underestimation for walking, cycling, and the 12 h EE, as well as an overestimation for sitting, standing, LPA, MPA, and VPA, when comparing FT to the criterion 2 (LOA: −3.93 to 4.71 kcal/min, Fig. 3). Moreover, an individualized underestimation for walking, cycling, LPA, and 12 h EE and an overestimation for sitting, standing, MPA, and VPA were observed, when comparing FP to the criterion 2 (LOA: −4.81 to 4.03 kcal/min, Fig. 4).

## Discussion

The current study consisted of two parts: a reliability and a validity protocol. We primarily found that Fibion wore on the thigh (FT) showed similar reliability to the Actigraph. However, low correlations between subsequent measurements with both devices indicated large random MEs, especially in low-intensity PAs (sitting). Furthermore, FT but not FP accurately detected different types (sitting and long sitting) and intensities (LPA, MPA, and VPA) of PA and EE (standing, LPA, MPA, VPA, and 12 h total EE), compared to both of the direct observation of the 12 h task guided sequence of tasks (criterion 1) and indirect calorimetry (criterion 2). But as such, FP differed significantly for all PA types and intensities (excepting LPA) when compared to both of the criteria, indicating that the location of the accelerometer is an essential factor.

The validity and reliability of ActiGraph have previously been well documented (Welk, Schaben & Morrow, 2004; Esliger & Tremblay, 2006; Vanhelst et al., 2012; Lee, Kim & Welk, 2014; Aadland & Ylvisåker, 2015). In line with the present results of the ActiGraph, FT provided relatively small marginal error by a low CV% (all <10%). However, the larger variance of ICCs (−0.165 to 0.609) indicated larger individual errors of the measurements for both FT and ActiGraph. The larger individual errors are likely due to the relatively shorter (i.e., 3 × 15 min) duration of our measurements compared to previous studies, which typically utilized durations of multiple hours up to several weeks (Aadland & Ylvisåker, 2015; Loprinzi & Smith, 2017; Hibbing et al., 2018; Arguello et al., 2018). Consequently, reliability design with longer duration may be further needed for Fibion.

Apart from the reliability test, the second aim of this study was to test the validity of the Fibion worn at two different positions (FT and FP), to determine different types, intensities of PA and EE throughout a simulated 12 h working day. Previous studies generally tended to use short protocols or isolated tasks for the validation of accelerometer-based gadgets (Duffield et al., 2004; Sun, Schmidt & Teo-Koh, 2008; Powell et al., 2016). However, shorter protocols are inevitability unable to capture the infinite number of activities in realistic free-living environments. Thus, our protocol provided a more advanced design to simulate extended periods of realistic activities of daily living with self-selected speeds and cadences, to reflect the current PA recommendations and to avoid excessive sitting (Buckley et al., 2015). Throughout this protocol, FT was found to provide a moderate differentiation for the duration of sitting, long sitting, LPA, MPA, and VPA. However, the LOA provided by the Bland–Altman plots were rather large, especially for FP. Moreover, the MEs appeared to be random, with low correlations being observed in sitting and standing tasks. As the magnitude of the ME seemed to be independent of the PA types, they were likely to be caused by the algorithm of the device rather than by movement artifacts, and this was also reported by other studies (Lee, Kim & Welk, 2014; Kooiman et al., 2015).

A unique feature of the present study was to compare the EE assessed by Fibion with that assessed by a portable gas analyzer during the prolonged simulated real-life protocol. Since the indirect calorimetry was used as a gold standard, we were able to define LPA, MPA, and VPA in an accurate manner, whereas, other studies used constant values for individual PA intensities (Adam Noah et al., 2013; Sasaki et al., 2015; Price et al., 2016; Lyden et al., 2017; Montoye et al., 2017). Interestingly, we did not find differences in standing, LPA, MPA, VPA, and 12 h EE between FT and oxygen consumption. This implied that the data of Fibion seemed to be valid, at least when compared to other methods of EE estimation. However, large differences were observed in the agreement between FT and PT, indicating poor agreements for the detection of PA types and intensities when Fibion was worn in the pocket (Fig. S1). It is possible that the Fibion devices did not consistently remain in the recommended location of the frontal part of the thigh, or participants wore rather loose shorts that allowed the device to shift inside the pocket. This indicated that the proper location of the accelerometer is essential for accurately assessing PA and EE for daily use.

When interpreting the findings of this study, one should bear in mind that the participants were relatively young and were selected as a convenience sample from a university and the nearby communities, which limited the generalizability of the results. Furthermore, walking and cycling were performed indoors in this study, and they might differ from the outdoor environments. Last, the laboratory-based setting of the validity study was also a limitation, given that the execution of tasks may differ between laboratory-based and free-living conditions, even though all efforts were made to simulate natural living conditions.

## Conclusions

Fibion located on the thigh (FT) provided similar reliability to that of the Actigraph. However, low correlations between subsequent measurements of both devices indicated large random MEs, which were somewhat diminished during the simulated 12 h real-life test. Furthermore, FT may accurately determine the types, intensities of PA and EE during prolonged periods with substantial changes in postures, indicating that the location of the accelerometer is essential. Further study with a large cohort is needed to confirm the usability of Fibion, especially for detecting the low-intensity PAs.

## Supplemental Information

10.7717/peerj.5775/supp-1 Supplemental Information 1 Table S1. 12-h guided sequence of simulated tasks of the validity protocol.

10.7717/peerj.5775/supp-2 Supplemental Information 2 Table S2. Definitions of activity codes (1 to 12), and explanations of how the codes were subsequently separated into five different types (sitting, long sitting, standing, walking, and cycling) and three intensities (LPA, MPA, and VPA) of PA. PA = physical activity.

10.7717/peerj.5775/supp-3 Supplemental Information 3 Fig. S1. Inter-individual differences of PA types (duration, min) for Sitting, Standing and Activity for Fibion in a thigh strap (FT) and Fibion in a pocket (FP). The “Activity” was defined as all activity types except sitting.

10.7717/peerj.5775/supp-4 Supplemental Information 4 Fig. S2. Wearing locations of the devices for reliability (a) and validity (b) protocol. (Photo by Tao Zhang).

10.7717/peerj.5775/supp-5 Supplemental Information 5 Raw data for reliability protocol.

10.7717/peerj.5775/supp-6 Supplemental Information 6 Raw data for validity protocol.

## Additional Information and Declarations

### Data Availability

The following information was supplied regarding data availability: The raw data are provided in the Supplemental Files.
