# Supplementary material for: Reliability and validity of a new accelerometer-based device for detecting physical activities and energy expenditure
Source: PeerJ. 2018 Oct 11;6:e5775. doi: 10.7717/peerj.5775 (PMC6186411; doi:10.7717/peerj.5775)
Supplement: Supplemental Information 1 [file peerj-06-5775-s001.docx]

**Supplemental Table 1.** 12-h guided sequence of simulated tasks of the validity protocol.

| Time | Activity code | Simulated task | Actual tasks performed |
| --- | --- | --- | --- |
| 07.20-07.40 | 0 | - | Measurement of resting metabolic rate |
| 07.40-8.00 | 2 | - | Eating breakfast |
| 8.00-8.20 | 6 | Making breakfast | Standing and walking a bit moving hands accordingly |
| 8.20-8.40 | 2 | Eating breakfast | Sitting and moving hands accordingly |
| 8.40-8.44 | 6 | Getting ready to go to work | Standing and walking a bit, moving hands accordingly |
| 8.44-8.45 | 7 | Walk from home to bicycle | Walking around in the lab |
| 8.45-9.00 | 10 | Commute to work (bicycle) | Simulated on ergometer |
| 9.00-9.01 | 7 | Walking from bike to office | Walking around in the lab |
| 9.01-9.30 | 2 | Working on computer (sitting) | Typing/surfing the web/reading from the screen (sitting) |
| 9.30-9.40 | 2 | Drinking break | Sitting, staying rather still |
| 9.40-10.00 | 5 | Working on computer (standing) | Typing/surfing the web/reading from the screen (standing) |
| 10.00-10.05 | 12 | Walking and climbing stairs | Stairs once up and down, rest of the time walking |
| 10.05-10.20 | 5 | Working on computer (standing) | Typing/surfing the web/reading from the screen (standing) |
| 10.20-10.35 | 2 | Working on computer (sitting) | Typing/surfing the web/reading from the screen (sitting) |
| 10.35-10.50 | 5 | Working on computer (standing) | Typing/surfing the web/reading from the screen (standing) |
| 10.50-11.00 | 2 | Working on computer (sitting) | Typing/surfing the web/reading from the screen (sitting) |
| 11.00-11.01 | 5 | Break (standing) | Standing |
| 11.01-11.30 | 2 | Working on computer (sitting) | Typing/surfing the web/reading from the screen (sitting) |
| 11.30-11.35 | 7 | Walking to lunch | Walking in the corridor next to lab and outside |
| 11.35-12.05 | 2 | Eating lunch (sitting) | Eating and relaxing (sitting) |
| 12.05-12.10 | 7 | Walking back from lunch | Walking in the corridor next to lab and outside |
| 12.10-12.25 | 5 | Working on computer (standing) | Typing/surfing the web/reading from the screen (standing) |
| 12.25-12.55 | 2 | Working on computer (sitting) | Typing/surfing the web/reading from the screen (sitting) |
| 12.55-13.00 | 5 | Walk | Walking in the corridor next to lab and outside |
| 13.00-13.30 | 2 | Working on computer (sitting) | Typing/surfing the web/reading from the screen (sitting) |
| 13.30-13.40 | 2 | Drinking break | Sitting, staying rather still |
| 13.40-13.55 | 5 | Working on computer (standing) | Typing/surfing the web/reading from the screen (standing) |
| 13.55-14.25 | 2 | Working on computer (sitting) | Typing/surfing the web/reading from the screen (sitting) |
| 14.25-14.35 | 2 | Talking on phone (sitting) | Holding phone on the ear |
| 14.35-14.37 | 7 | Walking to a meeting within office | Walking around in the lab |
| 14.37-15.22 | 2 | Having a meeting (sitting) | Sitting, watching video, handwriting sometimes |
| 15.22-15.42 | 2 | Eating a snack (sitting) | Eating and relaxing (sitting) |
| 15.42-15.57 | 6 | Giving presentation standing | Standing and moving a bit, moving hands accordingly |
| 15.57-16.20 | 2 | Having a meeting | Sitting, watching video, handwriting sometimes |
| 16.20-16.22 | 7 | Walking back from meeting | Walking around in the lab |
| 16.22-16.28 | 2 | Working on computer (sitting) | Typing/surfing the web/reading from the screen (sitting) |
| 16.28-16.32 | 2 | Drinking break | Sitting rather still |
| 16.32-16.40 | 5 | Talking on phone (standing) | Holding phone on the ear, standing and moving in place |
| 16.40-16.56 | 2 | Working on computer (sitting) | Working while sitting on a medicine ball |
| 16.56-16.57 | 5 | Standing | Standing next to the desk |
| 16.57-16.58 | 2 | Sitting | Sitting |
| 16.58-16.59 | 5 | Standing | Standing next to the desk |
| 16.59-17.00 | 2 | Sitting | Sitting |
| 17.00-17.01 | 7 | Walking from office to bicycle | Walking around in the lab |
| 17.01-17.16 | 10 | Commute home (on bicycle) | Simulated on ergometer |
| 17.16-17.17 | 7 | Walking from bicycle to home | Walking around in the lab |
| 17.17-17.50 | 2 | Dinner | Eating and relaxing (sitting) |
| 17.50-19.20 | 2 | Watching TV | Sitting or lying down on sofa |
| 19.20-19.30 | 6 | Changing clothes | Standing and moving |
| 19.30-19.40 | 8 | Exercise | Slower Walking (indoor track) |
| 19.40-19.50 | 9 | Exercise | Faster Walking (indoor track) |
| 19.50-20.00 | 11 | Exercise | Running (indoor track) |
