# Supplementary material for: Reliability and validity of a new accelerometer-based device for detecting physical activities and energy expenditure
Source: PeerJ. 2018 Oct 11;6:e5775. doi: 10.7717/peerj.5775 (PMC6186411; doi:10.7717/peerj.5775)
Supplement: Supplemental Information 2 — PA = physical activity. [file peerj-06-5775-s002.docx]

| Codes | Coded PA types | Combined PA types | Combined methods (codes) |
| --- | --- | --- | --- |
| 0 | Laying down | Sitting  Long sitting periods | 1-4  over 30 min sitting periods |
| 1 | Sitting legs up |  |  |
| 2 | Sitting |  |  |
| 3 | Sitting and MVC testing |  |  |
| 4 | Sitting on a gym ball |  |  |
| 5 | Standing | Standing | 5 |
| 6 | Standing and moving | Walking | 6-9 |
| 7 | Walking |  |  |
| 8 | Walking (slower exercise) |  |  |
| 9 | Walking (faster exercise) |  |  |
| 10 | Cycling | Cycling | 10 |
| 11 | Running | LPA | MET < 3 (Data from indirect calorimetry) |
| 12 | Climbing stairs | MPA | 3 < MET < 6 (Data from indirect calorimetry) |
| NA | NA | VPA | MET > 6 ( Data from indirect calorimetry) |

**Supplemental Table 2.** Definitions of activity codes (1 to 12), and explanations of how the codes were subsequently separated into five different types (sitting, long sitting, standing, walking, and cycling) and three intensities (LPA, MPA, and VPA) of PA. (PA = physical activity)
