# Supplementary figures and images for: Reliability and validity of a new accelerometer-based device for detecting physical activities and energy expenditure
Source: PeerJ. 2018 Oct 11;6:e5775. doi: 10.7717/peerj.5775 (PMC6186411; doi:10.7717/peerj.5775)

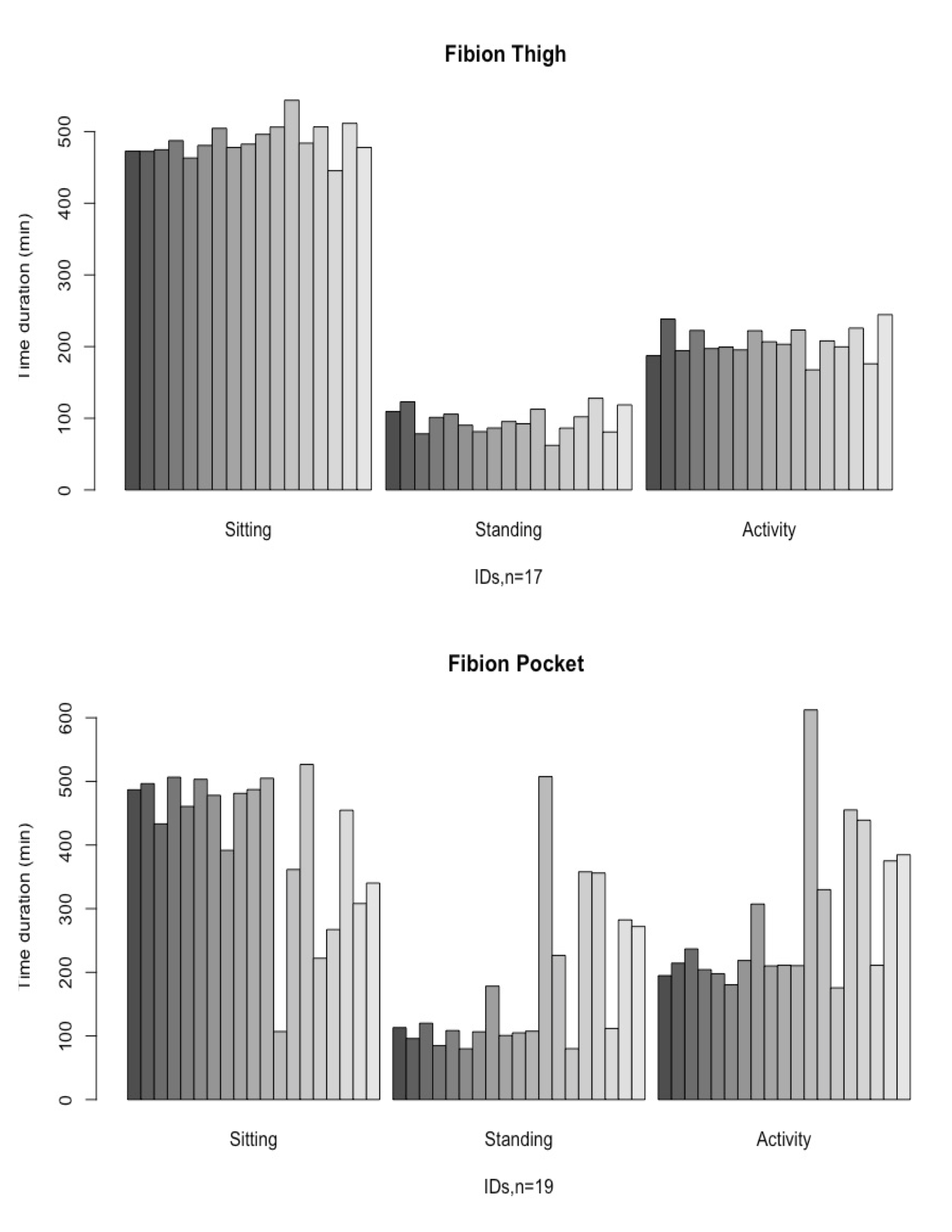

Supplement: Supplemental Information 3 — The “Activity” was defined as all activity types except sitting. [file peerj-06-5775-s003.png]

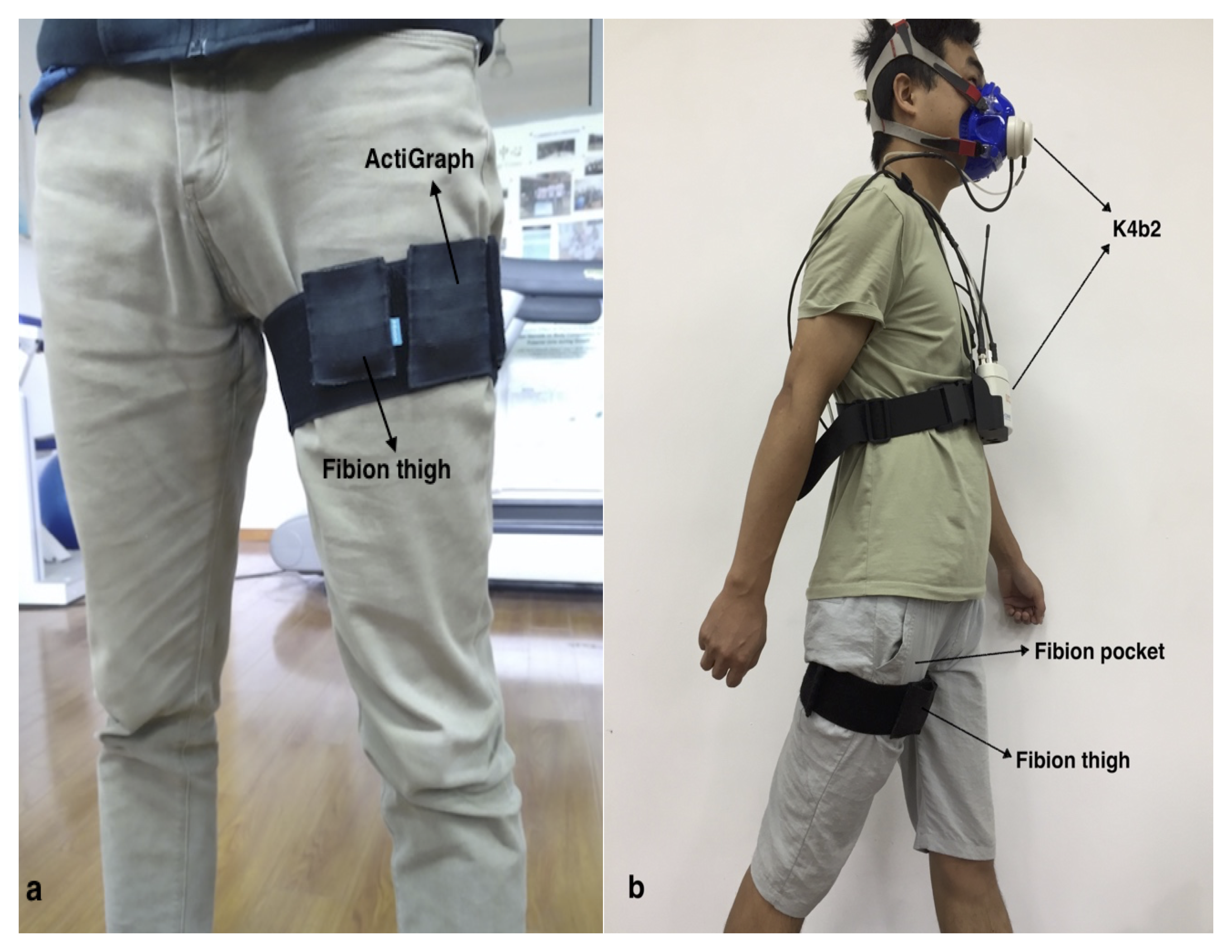

Supplement: Supplemental Information 4 — (Photo by Tao Zhang). [file peerj-06-5775-s004.png]
